# Evaluation of a Psychological Intervention for Patients with Chronic Pain in Primary Care

**DOI:** 10.3389/fpsyg.2017.00435

**Published:** 2017-03-23

**Authors:** Francisco J. Cano-García, María del Carmen González-Ortega, Susana Sanduvete-Chaves, Salvador Chacón-Moscoso, Roberto Moreno-Borrego

**Affiliations:** ^1^Departamento de Personalidad, Evaluación y Tratamiento Psicológicos, Universidad de Sevilla, SevilleSpain; ^2^Departamento de Psicología Experimental, Facultad de Psicología, Universidad de Sevilla, SevilleSpain; ^3^Departamento de Psicología, Universidad Autónoma de Chile, SantiagoChile; ^4^Centro de Atención Primaria Príncipe de Asturias, Servicio Andaluz de Salud, UtreraSpain

**Keywords:** formative evaluation, clinical effectiveness, chronic pain, Initiative on Methods, Measurement, and Pain Assessment in Clinical Trials (IMMPACT), methodological quality, primary care

## Abstract

According to evidence from recent decades, multicomponent programs of psychological intervention in people with chronic pain have reached the highest levels of efficacy. However, there are still many questions left to answer since efficacy has mainly been shown among upper-middle class patients in English-speaking countries and in controlled studies, with expert professionals guiding the intervention and with a limited number of domains of painful experience evaluated. For this study, a program of multicomponent psychological intervention was implemented: (a) based on techniques with empirical evidence, but developed in Spain; (b) at a public primary care center; (c) among patients with limited financial resources and lower education; (d) by a novice psychologist; and (e) evaluating all domains of painful experience using the instruments recommended by the Initiative on Methods, Measurement, and Pain Assessment in Clinical Trials (IMMPACT). The aim of this study was to evaluate this program. We selected a consecutive sample of 40 patients treated for chronic non-cancer pain at a primary care center in Utrera (Seville, Spain), adults who were not in any employment dispute, not suffering from psychopathology, and not receiving psychological treatment. The patients participated in 10 psychological intervention sessions, one per week, in groups of 13–14 people, which addressed psychoeducation for pain; breathing and relaxation; attention management; cognitive restructuring; problem-solving; emotional management; social skills; life values and goal setting; time organization and behavioral activation; physical exercise promotion; postural and sleep hygiene; and relapse prevention. In addition to the initial assessment, measures were taken after the intervention and at a 6-month follow-up. We assessed the program throughout the process: before, during and after the implementation. Results were analyzed statistically (significance and effect size) and from a clinical perspective (clinical significance according to IMMPACT standards). According to this analysis, the intervention was successful, although improvement tended to decline at follow-up, and the detailed design gave the program assessment a high degree of standardization and specification. Finally, suggestions for improvement are presented for upcoming applications of the program.

## Introduction

Pain is an unpleasant sensory and emotional experience associated with actual or potential tissue damage, or described in terms of such damage (Merskey, 1994). Pain becomes chronic when it loses its adaptive function, lasts longer than expected (3–6 months), and does not respond to the prescribed medical treatments. Pain and chronic pain are global, complex experiences for human beings, and interdisciplinary theoretical models have been developed to study them. One such model is the gate control theory (Melzack and Wall, 1967) and its more recent version, the neuromatrix theory (Melzack, 1999). Essentially, painful experience is defined at different levels here, including the sensory, behavioral, emotional and cognitive level, all of which are integrated in a more comprehensive framework of stress processes (for a more detailed description, see Gatchel et al., 2007). For this reason, psychology’s contribution to the study and treatment of chronic pain has been critically important for the past few decades.

Chronic pain is a public health issue in the developed world. In an aging population like that of Europe, 19% of the population suffers from chronic pain; in Spain, where this study was conducted, chronic pain stands at 11%. A recent study by Andrew et al. (2014) estimated the costs associated with chronic pain. In the work world, for every dollar lost by the average person, the costs associated with a person suffering from chronic pain are between $3.60 and $12.50 for absenteeism, between $2.50 and $3.00 for loss of productivity, and between $1.90 and $2.60 in paid unemployment. In terms of healthcare costs, for every dollar spent on other patients, the costs associated with a person suffering from chronic pain are between $2.50 and $3.00 in visits to primary care centers, between $3.30 and $7.60 in hospital stays, $4.00 in medicine and $3.00 in emergency care.

The gateway for patients with chronic pain in healthcare systems is usually the primary care center, as seen in Europe, where 70% of these patients saw a general practitioner (Breivik et al., 2006). Patients with chronic pain are seen as a challenging but low-priority customer similar to those suffering from mental health disorders, in contrast to high-priority patients like those suffering from cardiovascular disease (Johnson et al., 2013). Although professionals who see such patients usually have clinical practice guidelines, they tend not to use them to either evaluate or treat such patients because they are overwhelmed by the quantity and complexity of the demand. In most cases, such physicians limit themselves to prescribing drugs or referring the patient to a specialist.

There is unquestionable evidence on the efficacy of psychological intervention in chronic pain. According to the Society of Clinical Psychology (APA, 2016), evidence is particularly strong for two types of psychological intervention: cognitive-behavioral therapy (Morley et al., 1999; Huguet et al., 2014; Cherkin et al., 2016; Kroner et al., 2016) and Acceptance and Commitment Therapy (Veehof et al., 2011, 2016; Hann and McCracken, 2014). Other treatment options like relaxation therapy (Meeus et al., 2014), guided meditation and hypnosis have yielded moderate efficacy levels. Finally, evidence of efficacy has been growing for more recent treatment options such as eye movement desensitization and reprocessing (EMDR) (Tesarz et al., 2014) and particularly, mindfulness (Lauche et al., 2013). Given the current state of knowledge, multicomponent psychological treatments could be considered more efficacious than others and represent a viable alternative for healthcare when applied in small groups (APA, 2016). However, identifying efficacious treatment is one thing and getting the general population to benefit from such treatment is quite another. A good example of this is an epidemiological study conducted among 2,596 fibromyalgia patients in the USA: only 8% had received cognitive-behavioral therapy (Bennett et al., 2007).

In the scientific study of pain, the Initiative on Methods, Measurement, and Pain Assessment in Clinical Trials (IMMPACT) began in 2002 to improve the quality of assessments in clinical trials, bringing together scholars, regulatory bodies and public healthcare institutions, consumer and patient associations, and representatives from the pharmaceutical industry. Various scientific disciplines within healthcare like anesthesiology, clinical pharmacology, internal medicine, law, neurology, nursing, oncology, psychology, rheumatology and surgery are part of IMMPACT. The initiative has yielded three main results: the identification of the basic and complementary areas of the pain experience that must be evaluated (Turk et al., 2003; McGrath et al., 2008); the identification, development and validation of instruments to assess them (Dworkin et al., 2005; Turk et al., 2006; McGrath et al., 2008); and the determination of clinical importance standards to assess treatment outcomes (Dworkin et al., 2008, 2009; Turk et al., 2008).

The evidence presented above regarding both psychological treatment and the IMMPACT initiative is generally produced by studies conducted in ideal conditions, with the funding necessary for an adequate selection of participants: expert psychologists, patients with middle-high educational levels who are motivated to participate and do not leave the study, etc. In conditions such as these, many doubts regarding the efficacy of psychological intervention go unanswered. However, little information is available on clinical efficacy in real healthcare contexts: what if the studies focused on patients from a rural area in the south of Spain with different educational levels and from a different sociodemographic? What happens when they visit a primary care facility and are seen by a novice psychologist?

Ehde et al. (2014) addressed these challenges in an interesting review on cognitive-behavioral therapy for patients with chronic pain. The authors found only one study with rural and low literacy samples (Thorn et al., 2011). Worse still, they found no study that considered the level of experience of the therapist, but indicated that this variable might be relevant, since cognitive-behavioral therapy is more effective when performed by psychologists than other care providers (Nicholas et al., 2011).

These questions are what motivated us to assess a multicomponent cognitive-behavioral program specifically designed for patients with chronic pain, applied in a public primary care center located in the south of Spain, with participants from different socioeconomic and educational backgrounds and implemented by an inexperienced psychologist.

## Materials and Methods

### Participants

Patients at the Príncipe de Asturias primary care center participated in the study. The primary care center is located in Utrera, a small rural town in the province of Seville, Spain.

The inclusion criteria were the following: (a) to be at least 18 years old; (b) to have visited primary care due to difficulties handling chronic pain during the recruitment period (present maladaptive adjustment to pain); (c) to not be in the middle of an employment dispute or waiting for approval on a disability pension; (d) to not have a primary psychopathologic disorder; (e) to not be in psychiatric or psychological treatment, but could be taking psychotropic drugs; (f) the ability to follow group sessions, thus excluding conditions such as deafness, blindness, or dementia; (g) willingness to sign an agreement to attend the sessions (group and/or individual); and (h) not be hospitalized.

### Design

This study presents a quasi-experimental one-group pre-test – post-test – follow-up design (Shadish et al., 2002; Chacón-Moscoso et al., 2008). This means that there are three measurement instances: one before the intervention and two after the intervention (specifically, one immediately after the intervention and another 6 months later). Additionally, this design lacks a control group. As we are interested in studying the change over time in only one group, this is a within-subject design (APA, 2010).

### Variables and Measures

Initiative on Methods, Measurement, and Pain Assessment in Clinical Trials recommendations were used to assess the pain experience in terms of both procedures and instruments (Turk et al., 2003; Dworkin et al., 2005). The assessment covered pain, physical functioning, emotional functioning, and the patient’s rating of change. Although IMMPACT recommendations do not establish the main assessment variables, pain, specifically pain intensity, is usually considered a primary outcome. As a result, the remaining areas and variables would be considered secondary in this study, but also extremely important as indicators of possible improvements in the patients’ quality of life. To evaluate pain, the patient was asked to describe the intensity of perceived pain in the 24 h period preceding the interview and at the time of the interview, using a numerical scale with 0 meaning “No pain” and 10 meaning “Pain as bad as you can imagine” (Dworkin et al., 2005).

Physical functioning was evaluated through (1) the items *How much has pain interfered in your daily life during the last 24 h?* and *How much is pain interfering right now?*, with a four-point rating scale where 0 is nothing and 3, totally; and (2) the Spanish language version of the pain interference subscale (Ferrer et al., 1993) of the West Haven-Yale Multidimensional Pain Inventory (WHYMPI) (Kerns et al., 1985). The WHYMPI is the first psychometric instrument for multidimensional pain evaluation. The 11 items interference subscale consists of a seven-point Likert scale (0–6) to rate pain interference in daily life; the total points are then divided by the number of items. The psychometric properties of the original scale have been clearly demonstrated internationally (Haythornthwaite, 2003). Cronbach’s α was 0.68 for the Spanish language version of the interference scale (Ferrer et al., 1993).

Two instruments were used to evaluate emotional functioning: (1) the Profile of Mood States (POMS) (Haythornthwaite, 2004). This psychometric instrument assesses, using 58 adjectives rated from 0 (not at all) to 4 (extremely) on a five-point Likert scale, six mood states: Fatigue (0–28), Depression (0–60), Tension (0–36), Hostility (0–48), Confusion (0–28), and Vigor (0–32). In addition to six partial scores, it provides a global score on Total Mood Disturbance that ranges from -32 to 200 after adding the scores obtained in Fatigue, Depression, Tension, Hostility and Confusion, and subtracting the score obtained in Vigor. The POMS properties have also been demonstrated within the framework of IMMPACT with an internal consistency of the different scales between 0.63 (Confusion) and 0.96 (Depression) (Dworkin et al., 2005); and (2) the Beck Depression Inventory (BDI) (Beck et al., 1961). This instrument is comprised of 21 items that are answered on a four-point Likert scale (0–3). A total score is obtained by adding the values given for the 21 items ranging from 0 to 63. Higher values mean higher levels of depression. Specifically, 0–9 indicates none or minimal depression; 10–18 indicates mild to moderate depression; 19–29 indicates moderate to severe depression; and 30–63 indicates severe depression. This tool presents evidence of reliability and validity in the assessment of symptoms of depression and emotional distress (Dworkin et al., 2005).

The expected rating of change (pre-test) and the rating of change (post-test and follow-up) were evaluated using the Patient Global Impression of Change Scale (PGIC) (Guy, 1976). This measure is a single-item rating of a patient’s rating of improvement as the result of treatment on a seven-point scale that ranges from 1 “very much worse” to 7 “very much improved” with no change at the middle of the scale. Due to its simplicity, validity and reliability, the PGIC was included as a scale recommended by IMMPACT (Farrar, 2003).

Patient willingness was evaluated using the CONSORT (Consolidated Standards of Reporting Trials) guideline (Altman et al., 2001; Moher et al., 2001) which provides information on recruitment processes; the number of candidates excluded and the reasons for exclusion; the number of candidates who did not start treatment and the reasons; and the number of participants who abandon treatment and the reasons.

### Psychological Intervention

Psychological intervention consisted in a multicomponent protocol developed and published in Spain by a group of professionals and scholars, including one of the authors of this work (FJC). This protocol incorporates the principal cognitive-behavioral techniques with evidence of efficacy in pain treatment and combines them with a few others inspired by Acceptance and Commitment Therapy. A description of the program can be found in Moix and Casado (2011) and the full program is available at Kovacs and Moix (2011).

The program is structured in 10 weekly sessions, each lasting an hour and a half, that approach the following topics sequentially: (1) introduction to cognitive-behavioral intervention; (2) breathing and relaxation; (3) attention management; (4) cognitive restructuring I; (5) cognitive restructuring II; (6) problem-solving; (7) emotional management and assertiveness; (8) life values and goal setting; (9) time management and reinforcement activities; and (10) exercise, postural and sleep hygiene and relapse prevention.

Each session consists of three parts: first, a review of doubts and the tasks presented in the previous session; second, a discussion of the contents corresponding to the current session; and third, an overview of the tasks for the following session.

In addition to providing a handbook for the therapist, the program provides each patient with a dossier that includes a summary of the sessions and the tasks to accomplish as well as a CD audio guide on the breathing and relaxation exercises done in session 2.

The 40 patients assigned to the intervention were divided into three groups based on age and gender variables that will be detailed in Section “Results.” The first consisted of 14 women ages 33–55, the second of 13 men ages 33–55, and the last of 13 patients (eight women and five men) between ages 55 and 69. The total compliance rates for the full sessions were 78% in group 1 and 69% in groups 2 and 3. In groups 2 and 3, the intervention was not applied to two patients and in group 1, it was not applied to one patient; one patient from group 1, three from group 2 and two from group 3 discontinued.

### Procedure

This study was carried out in accordance with the recommendations of the Ethics Committee of the Southern Seville Health District (Andalusian Health Service) with written informed consent from all subjects. All subjects gave written informed consent in accordance with the Declaration of Helsinki. The protocol was approved by the South Seville Health District (Andalusian Health Service).

This study was carried out as part of a scientific-technical agreement with Southern Seville Primary Care. As part of this agreement, the second author of this study, MCG, then a post-graduate student, was selected through Ícaro^1^, a blog to manage practices in business and employment, as the psychologist who would carry out the study. She was selected because of an impressive academic record and after receiving a positive evaluation in a personal interview. The first author, FJC, informed her of the aim of the intervention and the task she was going to carry out; gave her all the materials (slides, handbook, dossier for the patients and CDs to be used during relaxation techniques); and provided her with training in a 4-h session.

The first step was to get the healthcare personnel, doctors and nurses involved in patient information and recruitment. This task that was handled by the last author of this study, RM. Recruitment relied on the inclusion criteria specified in Section “Participants.”

Following patient recruitment by the healthcare personnel, MCG informed the patients what the study entailed. The patients then signed the informed consent form and their first appointment was scheduled. During that appointment, each patient had a one-on-one interview with an undergraduate psychology student instructed in the application of the measures to be used in the study. Next, they participated in the group intervention with MCG. The sessions were held in a meeting room in the center with audiovisual equipment and mats for the participants to do the breathing and relaxation exercises.

Formative assessments (Chacón-Moscoso et al., 2013) were done throughout the process (before, during and after the implementation of the program). Immediately after the program ended and 6 months later, another assessment session with a one-on-one interview like the one described above was held.

All the data collected before the intervention, immediately afterward and 6 months later were anonymously added to a database by interning students from the authors’ departments and supervised by two of the authors, SS and SC, who also did the statistical analysis using the SPSS 22.

### Statistical Analyses

Cronbach’s (1951) α was used to test the reliability of the measures gauged with psychological tests and comprising more than one item, specifically the pain interference subscale of WHYMPI, POMS (subscales and global score), and BDI. Additionally, given the small sample size, in order to obtain a more precise reliability coefficient the unbiased estimator of Cronbach’s α was calculated (Feldt et al., 1987); and the significance of each unbiased estimator was calculated using the procedure of Kristof (1963) and Feldt et al. (1987). Following criteria established by George and Mallery (2003), values above 0.9 were considered excellent; between 0.8 (excluded) and 0.9 (included), good; between 0.7 (excluded) and 0.8 (included), acceptable; and between 0.6 (excluded) and 0.7 (included), questionable. Following criteria by Huh et al. (2006), values equal or higher than 0.7 were considered appropriate.

To study the changes to the different dependent variables across the three measurement instances (pre-test, post-test and follow-up), we first checked the normality assumption using Shapiro–Wilk’s test –*W*– (Shapiro and Wilk, 1965), adequate for small samples (*N* ≤ 50). When normal distribution was rejected (*p* ≤ 0.05), we used a non-parametric test (Friedman test); when this assumption was accepted (*p* > 0.05), we calculated a parametric test (ANOVA for repeated measures). In the case of ANOVA, Mauchly’s test of sphericity was calculated. When sphericity was assumed (*p* > 0.05), no correction of degrees of freedom *(df)* of *F* distribution was made; when it was rejected (*p* ≤ 0.05), *df* were multiplied by Greenhouse–Geisser’s epsilon to correct them.

Additionally, linear and quadratic trend contrasts were used to compare the three levels (pre-test, post-test, and follow-up). ANOVA trend analysis was used as a parametric test and showed results to be statistically significant when *p* < 0.05. As a non-parametric test, *post hoc* comparisons for trends were used (Marascuilo and McSweeney, 1967); here results were statistically significant when zero was not included in the interval obtained with a confidence level of 0.95. A significant linear trend would be interpreted as an increase, or at least maintenance, of changes detected in post-test during follow-up. In our case, this would be ideal. A significant quadratic trend would be interpreted as a reversal of the change detected in post-test during follow-up.

To calculate the effect size in the case of ANOVA, the partial eta or omega squared index can be overestimated in repeated measure designs (Olejnik and Algina, 2003). For this reason, we proceeded to calculate *r*^2^ by dividing the sum of squares of the intra-subject by the addition of the sum of squares of the intra-subject, the sum of squares of the intra-subject error and the sum of squares of the within-subject error. To calculate the effect size in the case of Friedman test, we calculated Kendall’s *W* coefficient of concordance, considered a strength-of-relationship index. It ranges from 0 to 1. Higher values indicate a stronger relationship (Green and Salkind, 2010). To interpret the effect size, we follow the conventional levels (Cohen, 1992) of effect size: small (0.01), medium (0.06), and large (0.16).

Finally, we used the IMMPACT clinical importance criteria (Dworkin et al., 2008). In terms of pain intensity, score drops (mean differences) between 1 and 2.9 were considered scarcely important; 3–4.9, moderately important; and above 5, substantial. In terms of the WHYMPI interference subscale, score drops equal to or higher than 0.6 are considered clinically important. For the POMS subscales, a reduction (or increase in the case of Vigor) of the score equal to or higher than two points is considered clinically important. In the case of the scale total, the required reduction is at least 10 points. Finally, in terms of the patient’s perception of improvement (PGIC), *minimally improved* (category 5) suggests a minor change, *much improved* (category 6), a moderately important change, and *very much improved* (category 7), a substantial change. In all cases, we compared the score obtained in pre-test with post-test and pre-test with follow-up.

## Results

Forty patients participated in the study. The age range was 33–69, with an average age of 47.9 and a standard deviation of 8.68. Twenty-two patients (55%) were women and 18 (45%) were men; 38 (80%) were married or lived with a partner; four (10%) were separated or divorced; three (7.5%) were single; and one (2.5%) was widowed. Eighteen (45%) had finished only elementary school and 10 (25%) had not; 11 (27.5%) had received their high school degree; and only one (2.5%) had attended college. In terms of employment, nine (22.5%) were unemployed; nine (22.5%) were housewives; 12 (30%) worked; eight (20%) had received early retirement for illness; one (2.5%) had retired after reaching retirement age; and one (2.5%) was laid off. According to their diagnoses, 22 (55%) were suffering from chronic low back pain, 12 (30%) from fibromyalgia and the remaining six (15%) from chronic headaches. They had been dealing with chronic pain for 2–30 years, with an average of 16.75 years and a standard deviation of 9.14 years. In 22 (55%) of the cases, the patient’s support person was their partner or spouse; in 13 (32.5%) of the cases, their father or mother; and in the remaining five (12.5%), other people. In 30 (75%) of the cases, the support person lived with the patient.

One important advantage of this intervention program is its high degree of standardization and specificity, aspects that facilitate its assessment and its replication and, as a consequence, allow its results to be generalized. Next we present the evaluation of the intervention program before, during and after the implementation.

### Before the Intervention: Needs Assessment, and Evaluation of Objectives and Design

In general, as this stage was based on IMMPACT recommendations, the objectives, design and instruments used to measure the aspects that the intervention aims to improve were all based on empirical evidence and a theoretical framework.

In order to facilitate the comparison with the results (measures before and after the intervention), information about the scores obtained by the sample before the intervention and its reliability are presented in Section “After the Intervention: Evaluation of Outcomes.”

The study of the internal coherence of the program yielded adequate results: all the needs had an associated objective, and at least one activity was included for each objective. Specifically, sessions 2 (training in breathing and relaxation) and 10 (physical activity, sleep and postural hygiene, and relapse prevention) were developed to reduce perceived pain; sessions 3 (attention management), 6 (problem solving), 8 (life values and goal setting), 9 (time management and reinforcement activities) and 10 were implemented to reduce the degree to which pain interferes in a patient’s life; sessions 4 and 5 (cognitive restructuring), 6, and 7 (emotions management and assertiveness) were developed in order to improve mood; and all activities (from session 1, the introduction to cognitive-behavioral intervention, through session 10) had a positive influence on patient’s perceived satisfaction. Additionally, the timeframe was realistic and the materials available for each activity were made explicit.

### During the Intervention: Evaluation of Implementation

**FIGURE 1:**
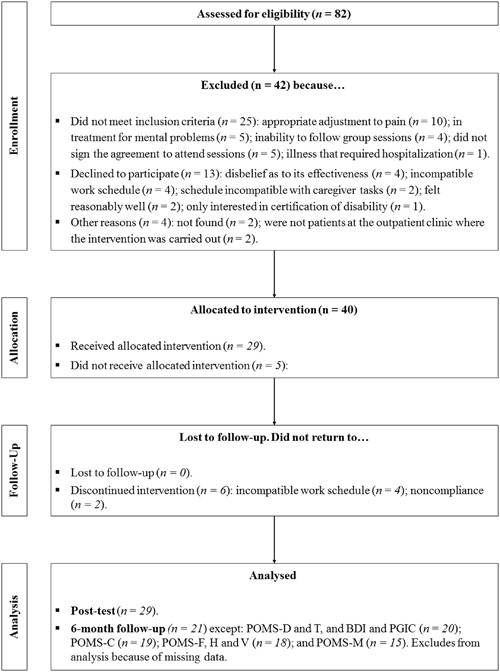
**Consolidated Standards of Reporting Trials (CONSORT) flow chart of participants through the study (Moher et al., 2001).** WHYMPI, West Haven-Yale Multidimensional Pain Inventory; POMS, Profile of Mood States; F, fatigue; D, depression; T, tension; H, hostility; C, confusion; V, vigor; M, total mood disturbance; BDI, Beck Depression Inventory; PGIC, Patient Global Impression of Change Scale.

As a measure of participant willingness, **Figure 1** presents a participant flow chart in keeping with CONSORT recommendations (Moher et al., 2001).

### After the Intervention: Evaluation of Outcomes

#### Reliability

**Table 1** presents the reliability results. All were significant at 95% CI. Considering the unbiased estimator of Cronbach’s α, six (22.2%) were excellent, 10 (37%) were good, nine (33.3%) were acceptable, and two (7.5%) were questionable (the subscale Tension of POMS in the pre-test and the follow-up). Overall, 25 (92.6%) of the results reached at least appropriate values (above 0.7) and the remaining two (7.5%) were close to 0.7 (concretely, 0.684 and 0.669).

**Table 1:** Reliability.

	Pre-test					Post-test					Follow-up				
	*α*	*N*	*ᾱ*	*F*	*p*	*α*	*N*	*ᾱ*	*F*	*p*	*α*	*N*	*ᾱ*	*F*	*p*
WHYMPI	0.728	40	0.796	4.902	<0.001	0.744	29	0.795	4.883	<0.001	0.765	21	0.922	12.766	<0.001
POMS-F	0.717	38	0.732	3.735	<0.001	0.800	29	0.814	5.385	<0.001	0.889	18	0.902	10.210	<0.001
POMS-D	0.845	38	0.853	6.820	<0.001	0.907	29	0.914	11.580	<0.001	0.878	20	0.891	9.161	<0.001
POMS-T	**0.666**	38	**0.684**	3.165	<0.001	0.782	29	0.798	4.940	<0.001	**0.630**	20	**0.669**	3.021	<0.001
POMS-H	0.829	38	0.838	6.182	<0.001	0.886	29	0.894	9.447	<0.001	0.705	18	0.740	3.842	<0.001
POMS-C	0.765	38	0.778	4.498	<0.001	0.766	29	0.783	4.602	<0.001	0.819	19	0.839	6.215	<0.001
POMS-V	0.701	38	0.717	3.536	<0.001	0.804	29	0.818	5.495	<0.001	0.739	18	0.770	4.342	<0.001
POMS-M	0.932	38	0.936	15.546	<0.001	0.960	29	0.963	26.923	<0.001	0.978	15	0.981	53.030	<0.001
BDI	0.876	38	0.883	8.525	<0.001	0.852	29	0.863	7.277	<0.001	0.860	20	0.875	7.983	<0.001

*WHYMPI, West Haven-Yale Multidimensional Pain Inventory; POMS, Profile of Mood States; F, fatigue; D, depression; T, tension; H, hostility; C, confusion; V, vigor; M, total mood disturbance; BDI, Beck Depression Inventory; *ᾱ*= unbiased estimator of Cronbach’s α. α and *ᾱ* lower than 0.7 are marked in bold.*

#### Normality

Considering the 14 variables and the three instances separately (14 × 3 = 42 combinations), the normality assumption using Shapiro–Wilk (*W*) was accepted on all occasions but nine: 24-h intensity, follow up (*W* = 0.857, *p* = 0.027); 24-h interference, pre-test (*W* = 0.639, *p* < 0.001) and follow-up (*W* = 0.798, *p* = 0.005); present interference pre-test (*W* = 0.639, *p* < 0.001) and follow-up (*W* = 0.849, *p* = 0.022); POMS-V, follow-up (*W* = 0.841, *p* = 0.017); BDI, pre-test (*W* = 0.834, *p* = 0.003); and PGIC post-test (*W* = 0.816, *p* = 0.008) and follow up (*W* = 0.851, *p* = 0.023).

As a result, the calculations for the six variables affected by normality rejection in at least one instance (24-h intensity, 24-h interference, present interference, POMS-V, BDI and PGIC), were done using non-parametric tests.

#### Effectiveness of the Psychological Intervention

##### Pain

**Table 2** presents the results. In terms of pain, both the pain intensity present at the time of the interview and the pain experienced in the 24 h beforehand diminished in a statistically significant manner after the intervention, with a large effect size.

**Table 2:** Global comparison at the different instances of measurement, trend contrasts and clinical significance when comparing pre-test to post-test and to follow-up.

Variable	Pre-test		Post-test		Follow-up		Global			^f^Clinical significance		Linear trend		Quadratic trend	
	*M*	*SD*	*M*	*SD*	*M*	*SD*	*N*	Statistic	ES	Pre–post	Pre-follow-up	Statistic	ES	Statistic	ES
24-h Inten	7.52	1.03	4.95	1.72	6.76	2.36	21	^b^19.9***	^d^0.47^+++^	2.57 ^♠^	0.76	^h^[-0.0140, 1.054]	—	^h^**[1.215, 3.065]**	—
Pr Inten	6.71	1.23	4.05	1.56	5	2.88	21	^a,c^10.37**	^e^0.3^+++^	2.66^♠^	1.71 ^♠^	^c^5.6*	^e^0.13^++^	^c^24.24***	^e^0.25^+++^
24-h Inter	2.43	0.55	1.52	0.91	2.24	0.89	21	^b^10.26**	^d^0.24^+++^	—	—	^h^**[**-0.224, 0.844]	—	^h^**[0.425, 2.275]**	—
Pr Inter	2.33	0.66	1.52	0.87	1.52	1.08	21	^b^14.06**	^d^0.34^+++^	—	—	^h^**[0.366, 1.434]**	—	^h^[-0.205, 2.275]	—
WHYMPI	4.12	0.73	3.33	0.74	3.7	0.86	21	^c^9.62***	^e^0.15^+++^	0.79^♠♠♠^	0.42^♠♠^	^c^5.08*	^e^0.06^++^	^c^14.62**	^e^0.13^++^
POMS-F	15.33	5.76	10	5.29	13.63	7.28	18	^c^4.59*	^e^0.1^++^	5.33^♠♠♠^	1.7^♠♠^	^c^0.75	^e^0.01^+^	^c^7.6*	^e^0.11^++^
POMS-D	22.65	10.96	14.40	11.05	20.70	16.46	20	^c^4.60	^e^0.07^++^	8.25^♠♠♠^	1.95^♠♠^	^c^0.428	^e^0.01^+^	^c^9.673**	^e^0.08^++^
POMS-T	19.15	4.74	15.20	7.01	17.05	9.15	20	^c^3.26	^e^0.05^++^	3.95^♠♠♠^	2.10^♠♠♠^	^c^1.63	^e^0.02^+^	^c^0.355*	^e^0.04^+^
POMS-H	20.06	8.01	14	9.41	16.50	12.55	18	^c^3.34	^e^0.06^++^	6.06^♠♠♠^	3.56^♠♠♠^	^c^2.241	^e^0.02^+^	^c^4.476*	^e^0.05^++^
POMS-C	13.47	5.89	9.89	5.42	11.95	6.75	19	^c^4.03*	^e^0.06^++^	3.58^♠♠♠^	1.52^♠^	^c^1.678	^e^0.01^+^	^c^5.818*	^e^0.05^++^
POMS-V	10.89	4.78	16.37	6.49	13.42	4.83	18	^b^12.10**	^d^0.32^+++^	-5.48^♠♠♠^	–2.53^♠♠♠^	^h^**[0.026, 1.094]**	—	^h^**[0.575, 2.425]**	—
POMS-M	81.40	36.81	47.33	41.17	73.33	52.77	15	^c^5.03*	^e^0.1^++^	34.07^♠♠♠^	8.07^♠♠^	^c^0.583	^e^0.01^+^	^c^8.548*	^e^0.14^++^
BDI	17.95	10.36	10.15	7.62	33.45	8.61	20	^b^31.3***	^d^0.78^+++^	—	—	^h^**[**-**1.634,**-**0.569]**	—	^h^**[1.475, 3.325]**	—
PGIC	5.00	0	6.20	0.70	5.85	0.93	20	^b^22.07***	^d^0.55^+++^	^g♠♠^	^g♠♠^	^h^**[0.386, 1.454]**	—	^h^**[0.655, 2.505]**	—

*24-h, 24-hour; Inten, intensity; Pr, present; Inter, interference; WHYMPI, West Haven-Yale Multidimensional Pain Inventory; POMS, Profile of Mood States; F, fatigue; D, depression; T, tension; H, hostility; C, confusion; V, vigor; M, total mood disturbance; BDI, Beck Depression Inventory; PGIC, Patient Global Impression of Change Scale. ^+^, small effect size (around 0.01); ^++^, medium effect size (around 0.06); and ^+++^, large effect size (around 0.16). Following Initiative on Methods, Measurement, and Pain Assessment in Clinical Trials (IMMPACT) recommendations, ^♠^, minimally important change; ^♠♠^, moderately important change; and ^♠♠♠^, substantial change.*

^a^Sphericity was not assumed (Mauchly’s test of sphericity *p* < 0.001). ^b^Friedman test. ^c^ANOVA *F* for repeated measures. ^d^Kendall’s coefficient of concordance *W*. ^e^*r^*2*^*. ^f^Mean difference. ^g^More detailed information can be consulted on **Table 3**. ^h^Non-parametric *post hoc* comparisons for trend, 95% confidence interval (statistically significant trends –zero excluded from the confidence interval are in bold).

*p < 0.05; **p < 0.01; ***p < 0.001.

In present intensity, the clinical significance was minimally important and both linear and quadratic trends were significant. The quadratic trend was stronger, however, with a large effect size, while the effect size for the linear trend was medium. This can be interpreted as a slight maintenance of results obtained in post-test at follow-up.

On pain intensity in the previous 24 h, we found a minimally important change when pre and post-test results were compared, and no change in the pre-test and follow-up comparison. The quadratic trend was statistically significant. This suggests that after the intervention, there was a decrease in 24-h pain intensity, but an increase 6 months later.

##### Physical functioning

The 24-h and present pain interference and the WHYMPI interference score diminished in a statistically significant manner after the intervention with a large effect size.

The clinical significance in WHYMPI was substantial in the pre–post comparison and moderately important when comparing pre-test and follow-up. The significant linear and quadratic trends with medium effect size revealed that, although there was a slight deterioration, the improvement continued in the follow-up period.

There was a statistically significant deterioration with regards to 24 h-interference in the follow-up period (significant quadratic trend). Nevertheless, the improvement in present interference continued in the follow-up period (significant linear trend).

##### Emotional functioning

In general, we can say that there was a statistically significant improvement in POMS and BDI. The effect size was medium/large in all the variables. In all cases, the clinical significance implied a substantial change when comparing pre-test and post-test. Additionally, the quadratic trend was statistically significant in all cases. This can be interpreted as an important deterioration in a comparison of the post-test and follow-up. Comparing the clinical significance at pre-test and follow-up, we find that the deterioration does not represent a return to the starting point in all the variables studied, because there is a substantial change in POMS-T, POMS-H, and POMS-V (with this last variable also showing a significant linear trend), and a moderately important change in POMS-F, POMS-D and POMS-M. Moreover, BDI also yielded a significant linear trend in favor of a possible maintenance of the results obtained.

##### Improvement perceived by the patient

Patient Global Impression of Change Scale shows that the improvement participants expected before the intervention was statistically lower than the subjective improvement perceived by the participants after the intervention, with a large effect size and a moderately important clinical change. This variable presents a statistically significant trend both linearly and quadratically, so it can be concluded that participants maintain their positive assessment when comparing post-test and follow-up.

In more detail, **Table 3** shows that, at post-test, all patients noted improvement, with more than half reporting a moderately important change and around one-third reporting substantial change (the maximum). At the 6-month follow-up, two patients reported that their chronic pain was similar to what it had been before the intervention. However, approximately half noted a moderately important improvement and one-fourth, substantial improvement. Overall, 90% of the patients stated that they had improved 6 months after the intervention.

**Table 3:** Improvement perceived by patients after the intervention in the Patient Global Impression of Change (PGIC) scale.

	Category	Post (*N* = 29)		Follow-up (*N* = 20)	
		*f*	%	*f*	%
No change	4	0	0	2	10
Minimally improved^♠^	5	3	10.3	4	20
Much improved^♠♠^	6	17	58.6	9	45
Very much improved^♠♠♠^	7	9	31	5	25

*Following Initiative on Methods, Measurement, and Pain Assessment in Clinical Trials (IMMPACT) recommendations, ^♠^, minimally important change; ^♠♠^, moderately important change; and ^♠♠♠^, substantial change.*

## Discussion

This study has provided additional evidence on the generalization of multicomponent interventions that have been already shown in other contexts (Morley et al., 1999; Veehof et al., 2011; Hann and McCracken, 2014; Huguet et al., 2014). While such interventions are usually implemented in English-speaking contexts, this paper presents an implementation in a Spanish rural area. While reported interventions are generally performed in a very controlled context, the sample of this study was selected from among users of a public health center who came in for a consultation. Participants in most studies are usually upper-middle class with a high educational level; 70% of participants in this intervention had a low educational level (complete or incomplete elementary) and 45% had no paid work and, as a result, low income. Finally, it is usual to find a limited number of domains of painful experience to evaluate interventions; in this case, we evaluated all the domains of chronic pain using instruments recommended by IMMPACT, i.e., to measure pain, intensity of perceived pain the previous 24 h and at the time of the interview (Dworkin et al., 2005). To measure physical functioning, we utilized the items referring to pain interference in daily life in the previous 24 h and at the time of the interview, and WHYMPI (Kerns et al., 1985). POMS and BDI were used to gauge emotional functioning. To measure perceived improvement after the treatment, PGIC was used.

Patient flow data were similar to those of other studies. Wetherell et al. (2011) carried out a randomized controlled trial comparing acceptance and compromise therapy with cognitive behavioral therapy in patients with chronic pain. They reported that 66% of patients were excluded from the recruitment, 12% of patients did not receive the intervention, and 16% of patients dropped out. Our percentages were 51, 12.5, and 21%, respectively. The principal reasons for exclusion and drop out of our study were similar to those reported by Wetherell et al. (2011): schedule incompatibilities, adverse life events and non-compliance.

In spite of the variants our study introduced to the standard intervention, the program assessment showed a high degree of standardization and specification owed to its highly detailed design (Kovacs and Moix, 2011; Moix and Casado, 2011). Moreover, the evaluation followed the IMMPACT recommendations, using instruments with tested psychometric properties. This facilitates the replication of the intervention and reinforces the results obtained. Second, there was a high degree of internal coherence. The same measures taken before the intervention were repeated immediately after and again 6 months later, using the same instruments. This comparison of the three instances facilitated the analysis of the change and provided evidence not only of the program’s effectiveness but also of the duration of the effects for a longer period of time. Each assessed need had at least an associated objective to be covered and each objective had at least one activity to be reached, and fitted timeframe and resources. Third, explicit selection criteria for participants were applied to all potential participants (Chacón-Moscoso et al., 2016). Forth, the measures presented sufficient reliability coefficients. Fifth, we found evidence of effectiveness, as there was a statistically significant improvement after the intervention or at least a medium effect size in all the variables measured and all the domains taken into account; and substantial clinical change in 75% of the variables measured.

From our point of view, the main contributions of the study is to demonstrate that cognitive-behavioral therapy can be effective even if performed by an inexperienced therapist to groups of low-literacy patients with a low socioeconomic status. As for therapist experience, although common sense suggests that it should improve the effectiveness of therapy, the first longitudinal study that addresses this question, with data from 170 psychotherapists and 6,591 patients (Goldberg et al., 2016), did not endorse this. In our opinion, the highly structured intervention program and the wealth of resources and material available to the therapist minimize the possible impact of their inexperience. In terms of the second aspect, literacy and socioeconomic resources are considered a barrier for the efficacy of cognitive behavioral treatment of chronic pain (Campbell, 2011) and this led to the creation of personalization initiatives for these patients (Thorn et al., 2011; Eyer and Thorn, 2016). Even so, in the first study with personalized treatment (Thorn et al., 2011), 26.5% of patients did not complete the intervention, which is 5.5% more than in our study. This could be explained by the therapist’s familiarity with the patients and by the effort that she carried out to make the program contents understandable for the patients.

The improvement observed just after the intervention worsened in approximately two-thirds of the variables measured (only the quadratic trend was statistically significant), though the measures did not return to their starting points. The ostensibly mild deterioration is still strong enough to be statistically significant. Maintaining the long-term effects of these programs is another major challenge, considering the high chronicity of these patients (in our study, patients had been suffering from chronic pain for over 16 years on average). A possible moderating factor could be the quantity and quality of homework, a neglected aspect of cognitive behavioral therapy research, the importance of which has been revealed in a recent meta-analysis (Kazantzis et al., 2016). Anyway, it would be highly advisable to add some sessions after the intervention, one every 4 months, to maintain the improvements patients have obtained.

On the other hand, the principal limitation was the absence of a control group that would have enhanced the design and increased evidence of the intervention’s effectiveness. Nevertheless, a control group would not have been feasible in this study, because we were ethically obliged to offer the intervention program to every patient in a public primary care setting. In any case, we were less interested in the program efficacy than in identifying who could benefit from the intervention.

Further research is going to take two directions. First, we are going to adapt the intervention to a broader potential population. People with a disability such as deafness, blindness or dementia were excluded from the initial intervention, but we trust that it is possible to adapt the intervention to cases such as these. Second, in order to increase the evidence of the efficacy of the intervention applied in this study (Moix and Casado, 2011), a meta-analysis will be developed. This will assist us in obtaining a global effect size after a statistical synthesis of the results obtained in the different interventions while also allowing us to detect possible moderator variables that influence the effectiveness of these interventions. From this study, we would be able to establish practical recommendations for psychologists to increase the likelihood of success of this kind of programs.

## Author Contributions

FC-G came up with the initial idea and design. RM-B recruited the sample. MG-O carried out the intervention. SS-C and SC-M performed the analyses and interpreted the data. FC-F, SS-C, and SC-M were entrusted with drafting the manuscript. All the authors reviewed the manuscript, approved the final version to be published, and agree to be accountable for all aspects of the work, ensuring that questions related to the accuracy or integrity of any part of the work are appropriately investigated and resolved.

## Conflict of Interest Statement

The authors declare that the research was conducted in the absence of any commercial or financial relationships that could be construed as a potential conflict of interest.

## Footnotes

1 https://icaro.ual.es/
